# Chemical-Free Technique to Study the Ultrastructure of Primary Cilium

**DOI:** 10.1038/srep15982

**Published:** 2015-11-02

**Authors:** Ashraf M. Mohieldin, Wissam A. AbouAlaiwi, Min Gao, Surya M. Nauli

**Affiliations:** 1 Department of Biomedical & Pharmaceutical Sciences, Chapman University School of Pharmacy, Irvine, CA 92618; 2 Department of Urology, University of California at Irvine Medical Campus, Orange, CA 92868; 3 Department of Medicinal & Biological Chemistry, University of Toledo School of Pharmacy, Toledo, OH 43614; 4 Liquid Crystal Institute, Kent State University, Kent, OH 44242.

## Abstract

A primary cilium is a hair-like structure with a width of approximately 200 nm. Over the past few decades, the main challenge in the study of the ultrastructure of cilia has been the high sensitivity of cilia to chemical fixation, which is required for many imaging techniques. In this report, we demonstrate a combined high-pressure freezing (HPF) and freeze-fracture transmission electron microscopy (FFTEM) technique to examine the ultrastructure of a cilium. Our objective is to develop an optimal high-resolution imaging approach that preserves cilia structures in their best natural form without alteration of cilia morphology by chemical fixation interference. Our results showed that a cilium has a swelling-like structure (termed bulb), which was previously considered a fixation artifact. The intramembrane particles observed via HPF/FFTEM indicated the presence of integral membrane proteins and soluble matrix proteins along the ciliary bulb, which is part of an integral structure within the ciliary membrane. We propose that HPF/FFTEM is an important and more suitable chemical-free method to study the ultrastructure of primary cilia.

Primary cilia have been intensively studied over the last few years because they are relevant to a group of diseases called ciliopathies. Ultrastructurally, primary cilia are supported by microtubules and enclosed by the ciliary membrane. Each cilium has a bulging structure, known as a bulb, which was initially observed in 1959^1^. It was proposed that the ciliary bulb contained substances to be transported within the cilium and that it might represent a circumscribed region of the ciliary membrane^2^. Nonetheless, the ciliary bulb or swelling was always thought to be a passive reaction or response to changes in environmental osmotic pressure. This assumption was made despite the fact that the ultrastructure of a non-motile primary cilium had never been studied with transmission electron microscopy (TEM) to analyze its nanoscale structural detail.

The ciliary structure exhibits high sensitivity to chemical fixation. It has been known since the early 1960 s and confirmed in the 1990 s that chemical fixation during sample preparations for cilia analysis will result in fixation artifacts, including those involving the ciliary bulb structure^1^,^2^,^3^,^4^. Thus, a successful technique to study cilia requires the sample to be fixed very rapidly in their living state and without the involvement of chemicals and lengthy processing. However, such a requirement is not compatible with some of the widely used imaging techniques in order to obtain reliable results that are consistent with the native structure^5^.

To overcome this challenge in studying the ultrastructure of primary cilia, we have since established a freeze-fracture transmission electron microscopy (FFTEM) technique to examine the morphology of the cilia by abrupt high-pressure freezing (HPF). Our method consists of growing the cells directly in the HPF carriers, which will prevent the structural changes before and after sample preparation. The HPF/FFTEM technique is substantially different from other techniques that require chemical fixations. The HPF/FFTEM technique is a chemical-independent technique that uses rapid freezing and solution-free sample preparation.

The overall protocol for the HPF/FFTEM procedure is straightforward (Fig. 1). While some of the primary cilia are more obvious in one preparation compared with others (Supp Fig. 1), membrane fractures may not necessarily produce primary cilia (Supp Fig. 2). During the fracture of the cells, many fracture configurations are possible (Fig. 2). In most cases, however, a greater magnification is required to confirm the structure of a cilium. The position of the cilium also dictates the clarity of the image. For example, the grid of the sample support mesh and/or the replica image of the granular cell body could potentially hinder capturing and analyzing the entire cilium. In a different case, the cilium (or microvilli) may not have been replicated throughout the entire ciliary shaft. We term this “angled cilium”, in which only a short replica image of the cilium is observed. An optimal image of a cilium can be obtained when the cilium lays flat on the replica plate, and we call this “flat cilium”.

Many flat cilia have bulging structures (Fig. 3). These bulging structures or bulbs can have spherical or irregular shapes. The size of the replicated bulbs can range from 80 nm to approximately 1 μm. The biological functions of these bulbs were recently studied^5^. The significance of the bulb size, however, is still not clear. It is possible that the small bulbs result from a shallow fracture. However, various sizes of the bulb have also been observed during live-cell imaging, indicating that a cell may have the means to regulate the size and appearance of the bulb^5^. Live imaging of cilia can certainly circumvent the concerns of chemical fixation, but image resolution is limited to the wavelength of light used to observe the nanostructure of a cilium. Thus, the use of an electron microscope is preferred for the study of the singularity and nanostructure of a cilium.

Our results indicate that the ciliary bulb shares an intact structure along with the ciliary shaft, and there was no indication that the ciliary bulb is attached separately to the outer ciliary membrane. It is apparent that the bulb bilayer is part of the cilia bilayer. Furthermore, the HPF/FFTEM images show particle indentions along the ciliary membrane, including the ciliary bulb. In a replica of any cell membrane, scattered particles are often visible, indicating the existence of integral membrane proteins. This finding therefore suggests that the ciliary bulb and cilium itself contained integral and/or matrix proteins.

The main challenge with other TEM approaches is the detrimental effect of chemical fixative on cilia structure. Many fixatives, including 4% paraformaldehyde, could dramatically alter ciliary morphology (Fig. 4). In general, shorter and imbalanced cilia are observed when they are chemically fixed. The grossly damaged structure of the cilium makes it extremely difficult to obtain a nicely flat cilium for imaging purposes. We therefore believe that the challenge with many TEM approaches is the inability to obtain cilia with excellent structural integrity.

The freeze-fracture technique was first discovered in the 1950 s for the preparation of samples for electron microscopy^6^,^7^. Hall and Meryman introduced the sublimation of ice to reveal surface structures by using the combination of freezing and etching techniques. Over the years, the technique started to have more impact on the study of biological ultrastructure^8^. The effectiveness of this technique was further demonstrated when freeze fracture was performed in yeast cells to reveal the three-dimensional ultrastructure of the specimen^9^,^10^. Since then, the freeze-fracture technique has been a very useful tool in studying the ultrastructure of lipid structures, fats and oils, membrane lipids, non-lipid lamellae, dry lipids thermotropic states, lyotropic states, lateral phase separation in lipid bilayers and biological membranes, non-lamellar lipid structures, single micelles, aggregates of micelles, bicontinuous cubic phases of type II, biological membranes and surface views or membrane splitting^8^.

The HPF/FFTEM technique is certainly appropriate for all of these studies. Furthermore, we believe that the HPF/FFTEM technique is particularly suitable for studies associated with cytoskeletal-based cellular structures, such as microvilli, kinocilia, photoreceptor cilia, motile and non-motile cilia, and various junctional proteins or receptors whose structural activities depend on intermediate filaments. Specifically, cytoskeletal proteins and intermediate filaments are relatively stable structures under physiological conditions. However, open filaments have been observed after chemical fixation. More surprisingly, even changes in buffer solution on the electron microscope grids could have very pronounced effects on intermediate filaments. These properties have drawn attention to the fact that intermediate filaments are very sensitive to changes in the extracellular microenvironment, as predicted by many biologists and microscopists in the field^11^.

Our study, examining the nanostructure of the ciliary membrane using a chemical-free technique, is only a beginning. With rapid development of new ways to adopt the freeze-fracture technique and accessibility to image-reconstruction, we could potentially obtain 3D topology, cytoskeletal structure and protein localization within a cilium using TEM in the near future. Certainly, immunogold protein localization can be achieved with freeze-fracture replica. This method used to be very time-consuming, requiring five days to prepare one group of samples because the freeze fracturing allowed retrieval of only one of the two membrane fracture faces. With recent advancements in methodology, freeze fracture replica immunogold-labeling in biological samples has overcome many of the challenging issues associated with two apposed membrane fractures. The new approach offers an unambiguous identification of the membrane side and may become a standard and more reliable procedure^12^. Furthermore, freeze fracture can also be used to study cytoskeletal proteins. Coupled with deep etching or freeze etching, freeze fracture has been used to capture both microtubules and actin filaments^13^,^14^. Without doubt, the relatively very old procedure of freeze fracturing offers easy access to the interiors of samples that can otherwise only be reached by exceedingly difficult procedures with chemical fixation. The visibility of the ciliary membrane with freeze fracturing certainly provides a unique angle and is more advantageous.

A wide range of variations of FFTEM-related techniques has also been developed over the past half century and is still widely used for biological materials and some other soft-matter materials, such as liquid crystals. FFTEM is often considered to be a traditional or even “old” technique compared with some of the new developments, such as cryo-EM of vitreous sections (CEMOVIS) and freeze substitution. However, some of the advantages that FFTEM can provide are often found to be unique and no other alternative technique is comparable. For example, its unique surface sensitivity was found to be extremely useful in studies of complex liquid crystal systems^15^. For biological materials, one of the strengths of FFTEM is that the frozen membranes may tend to fracture along the central hydrophobic core, which makes FFTEM a valuable tool for the study of cilia. On the other hand, CEMOVIS and freeze substitution are both ultramicrotomy-based techniques. It is generally difficult to obtain similar information without extra effort, such as serial sectioning and 3D reconstruction.

Of note is that many other FFTEM techniques require various chemicals and cryoprotectants that can deteriorate the biological samples easily. On the other hand, other cryo-TEM techniques use plunge-frozen thin solution films and require blotting of the excessive solution with filter paper. In addition to a shorter sample processing time, the HPF/FFTEM technique is thus superior by avoiding the use of cryo-TEM and chemical fixation throughout the process. Like other imaging techniques, a high level of expertise is needed to implement the HPF/FFTEM protocol. However, the HPF/FFTEM protocol is simple enough that if trained properly, a new trainee should be able to perform the technique independently in a very short time.

In summary, we suggest that the ultrastructure of the mammalian primary cilium is best studied with no chemical fixative. The use of the HPF/FFTEM approach offers various advantages to the study of the ultrastructure of a cilium. Technically, the same cryo-fixation procedure can also be used for other TEM techniques, namely CEMOVIS (or cryo-TEM on thin specimens prepared by focused ion beam) and freeze substitution. Compared with the latter two, the HPF/FFTEM technique provides a much simpler process and is more focused on the membrane structure and ultrastructure of primary cilia. HPF/FFTEM also involves an inexpensive procedure to reveal the ultrastructure of the ciliary bulb without the use of any chemical fixation and to maintain the cilia morphology in its best natural form. This technique is therefore an indispensable tool to study the etiology of cilia-related diseases by offering high-resolution of the ultrastructure image of primary cilia. We propose that HPF/FFTEM is an important and more reliable chemical-free method for studying the ultrastructure of primary cilium, which is sensitive to chemical fixation.

## Materials and Methods

### Reagents

1. Porcine kidney epithelial cells (LLC-PK1), or any cultured cells.
2. Phosphate buffer saline (PBS, pH 7.4) (*Fisher, Inc*).
3. Modified Eagle Medium (DMEM) (*Corning*).
4. Fetal bovine serum (FBS) (*HyClone, Inc*).
5. Penicillin/streptomycin (*Corning Cellgro*).
6. Formvar (*Electron Microscopy Science*).
7. Ethylene dichloride (*VWR*).
8. Collagen (*Corning*).
9. Trypsin (*Fisher, Inc.*)
10. Ethanol (*Fisher, Inc.*).

### Equipment

1. Cell Culture Incubator (*Sanyo* MCO-19AIC).
2. Freeze-fracture ring for the freeze-fracture carrier (*Leica* 16706867).
3. Freeze-fracture carrier for Leica EM PACT2 high-pressure freezer (*Leica* 16706866).
4. Freeze-fracture bayonet pod to lock carrier and ring (*Leica* 16707829).
5. High-pressure freezer with rapid transfer system (*Leica* EM PACT2).
6. Freeze-fracture apparatus (*BalTec*; BAF060).
7. Transmission electron microscope (FEI Tecnai F20 operated at 200 KV).
8. Forceps (VWR, 82027-384).
9. Tissue culture dish (35 mm; Biolite).

### Step-by-step protocol

1. Solution preparations (45 minutes).
  - Preparing Formvar solution.
    - Prepare 2% Formvar solution by dissolving 0.9 g of Formvar powder in 45 mL of ethylene dichloride.
    - Incubate solution in 37 °C water bath.
    - Shake solution vigorously if not dissolved.
  - Preparing collagen solution.
    - Prepare 50 μM collagen solution by dissolving collagen in cold PBS solution.
    - Collagen should dissolve easily.
    - Check and adjust pH to 7.4 if not dissolved.
  - These solutions are for cell culture use only. No additional solution is needed for HPF/FFTEM sample preparation and processing.
2. PAUSE POINT:
  - Formvar and collagen solutions can be stored for one week.
  - Formvar solution can be stored at room temperature.
  - Collagen solution should be stored at 4 °C.
  - Do not freeze Formvar and collagen solutions.
3. Preparing the gold-plated flat carriers for cell growth (60 minutes).
  - Immerse the gold-plated flat carriers in 95.0% ethanol for 5 minutes.
  - Wash the gold-plated flat carriers with sterile PBS three times under the cell culture hood.
  - Carriers can be further sterilized under ultraviolet light for 30 minutes.
  - Immerse the gold-plated flat carriers in 2% Formvar solution for 3–5 seconds.
  - Place the gold-plated flat carriers in small petri dish and let it dry for 10 minutes.
  - Add 50 μM collagen directly into the gold-plated flat carriers and let it dry for 10 minutes.
  - Wash the petri dish containing the gold-plated flat carriers with sterile PBS two times to wash out excess collagen solution.
  - Place the petri dish containing the gold-plated flat carriers under ultraviolet light for 30 minutes to sterilize the carriers.
  - Use sterile technique from here on.
4. PAUSE POINT: The carriers can be kept for 2–4 days at 4 °C.
5. Growing the LLC-PK1 cells on the gold-plated flat carriers (15 minutes; 24 hours).
  - Wash grown 60–70% confluent LLC-PK1 cells with PBS two times and add appropriate amount of trypsin.
  - Incubate cells in incubator for approximately 3 minutes.
  - Collect cells and remove trypsin by briefly centrifuging the cells in a test tube.
  - Add DMEM media containing 10% FBS and 1% penicillin/streptomycin.
  - Gently disperse cells.
  - Add approximately one million cells directly onto each of the gold-plated flat carriers.
  - Incubate cells at 37 °C in 5% CO_2_ for 24 hours.
  - At this stage, cells should reach approximately 70–80% confluence.
6. CRITICAL STEP (24–72 hours).
  - A long, well-developed cilium is easier to image and analyze.
  - Cilia growth can be induced through cell differentiation.
    - First Option:
      - Use DMEM containing 2% FBS.
      - Recommend 2% FBS to prevent cell death.
      - Recommend withdrawing penicillin/streptomycin to accelerate cell differentiation.
      - Grow cells for additional 24–48 hours.
    - Second Option:
      - Use cell-cell contact inhibition to promote cell differentiation.
      - Grow cells for additional 48–72 hours.
  - Note that cells on the gold-plated flat carriers can only be observed using upright or surgical microscope. Alternatively, to ensure cell growth on the gold-plated flat carrier, one of the gold-plated flat carriers can be transferred into a clean petri dish and incubated in trypsin to observe detached cells under a regular cell culture microscope.
7. Preparing cells for HPF/FFTEM (90 minutes for sample preparation; 8–24 hours for image analysis).
  - With live cells grown inside gold-plated flat carriers (*Leica* 16706866), carefully remove once carrier from the petri dish with a forceps.
  - Place a ring (*Leica* 16706867) on top of the carrier.
  - Load the carrier-ring assembly into a bayonet pod (*Leica* 16707829).
  - Tighten the diamond locking screw of the bayonet pod (on the side of the ring) so that the carrier assembly is securely sealed together.
  - Quickly transfer the bayonet pot into the Leica EM PACT2 high-pressure freezer for the rapid cryo-fixation.
  - Apply a high pressure (~2,000 bar) to the sample.
  - CRITICAL STEP: use 2,000 bar pressure to significantly prevent the nucleation and growth of ice crystals. Note that ice crystals could induce a sudden imploding of cell membrane and membrane rapture.
  - Set the sample stage to −165 °C inside the vacuum chamber.
  - During the pressurized process, inject liquid nitrogen rapidly onto the sealed assembly for the vitreous freezing.
  - After successful freezing, quickly transfer the assembly into a BalTec BAF060 freeze-fracture apparatus.
  - A successful freezing can be determined based on the relative changes of both measured pressure and temperature.
  - Note: We could simply read the measured pressure and temperature values from the instrument to ensure that the system is operating properly. If high-pressure freezing is properly applied and monitored from the instrument, the use of cryoprotectant can be avoided to prevent its non-specific chemical toxicity to the cells^16^. High-pressure freezing has the potential to freeze biological samples at 500 μm depth without apparent ice-crystal artifacts^17^.
  - Employ a built-in microtome to knock off the ring to fracture the frozen sample inside the carrier.
  - Perform the fracture immediately by the replication of the surface topography, which is performed by depositing a thin (~4 nm) Pt/C layer at 45° and a continuous carbon film (~20 nm) at 90°.
  - Take the sample to the airlock and then move outside.
  - Put the carrier into distilled water to dissolve the culture media.
  - Collect the suspended pieces of the replica by holey lacey carbon coated TEM grids.
  - Observe sample using a Tecnai TF20 field emission TEM operated at 200 KV (8–24 hours for image analysis).
  - Look for a typical fracture configuration for a cilium.

## Additional Information

**How to cite this article**: Mohieldin, A. M. *et al.* Chemical-Free Technique to Study the Ultrastructure of Primary Cilium. *Sci. Rep.* **5**, 15982; doi: 10.1038/srep15982 (2015).

## Supplementary Material

Supplementary Data

## Figures and Tables

**Figure 1:**
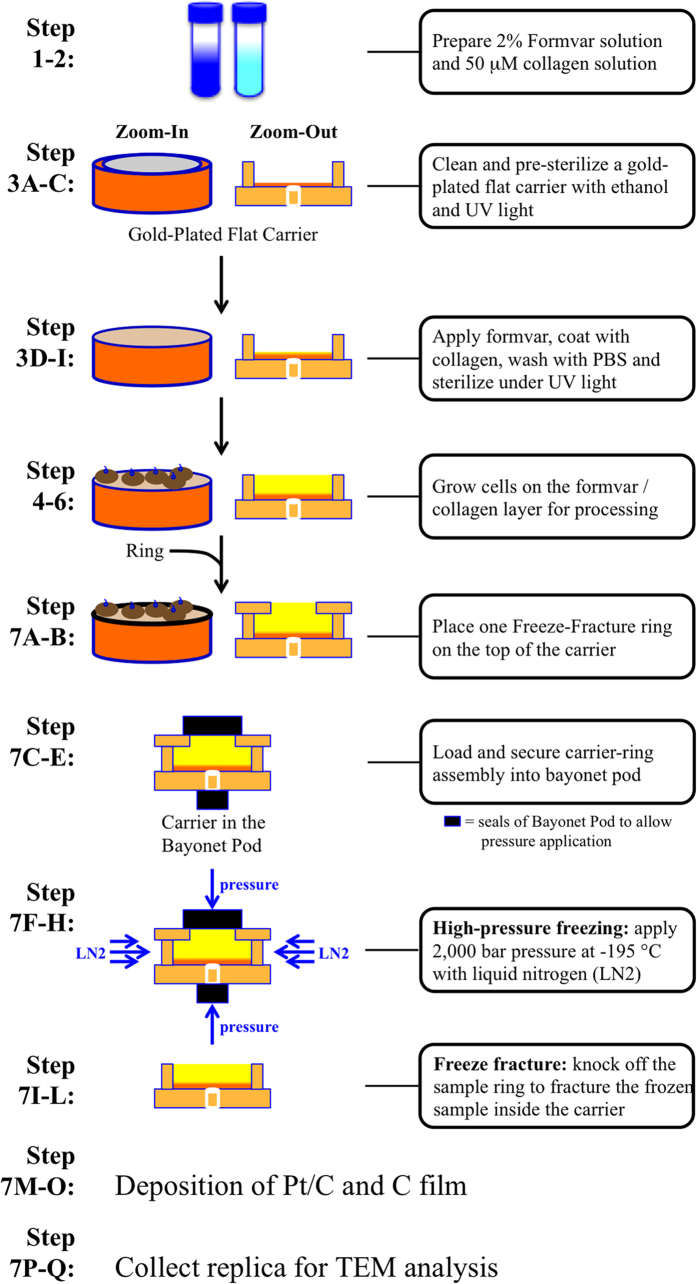
Schematic of experimental procedure. Schematic diagram depicting the HPF/FFTEM procedure used in the cilia study. Each of the steps represents a key procedural step and is described in more detail in the *Materials and Methods* section. Note that the schematic cartoon does not reflect the actual products and mechanics of the instrument.

**Figure 2:**
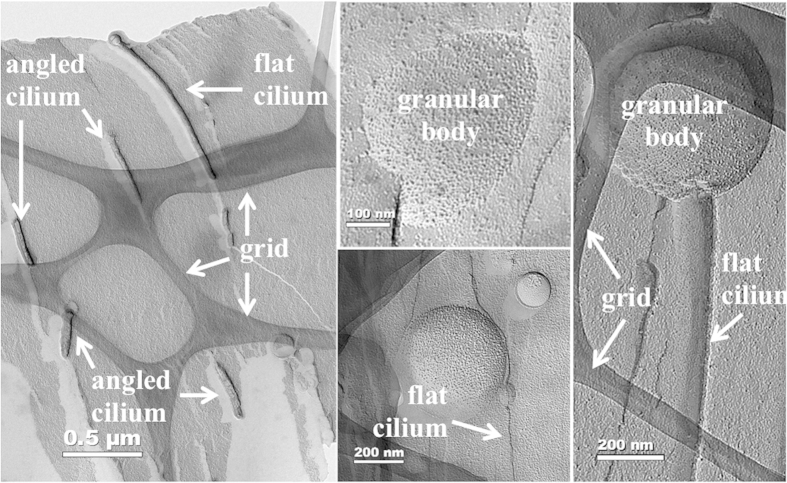
Typical FFTEM/HPF for cilia analysis. Shown here are some examples of FFTEM/HPF images; some preparations are cleaner than others. Clean preparations for cilia study are those with less granular body. The grid of sample support mesh can also be seen. Incomplete replica image of cilium (angled cilium) can be due to a less optimal angle or position of the cilium during fracture imprint, or it may represent short microvilli. A cilium laying flat on the surface during fracture and imprint would generate a more complete length of cilium, which is termed flat cilium.

**Figure 3:**
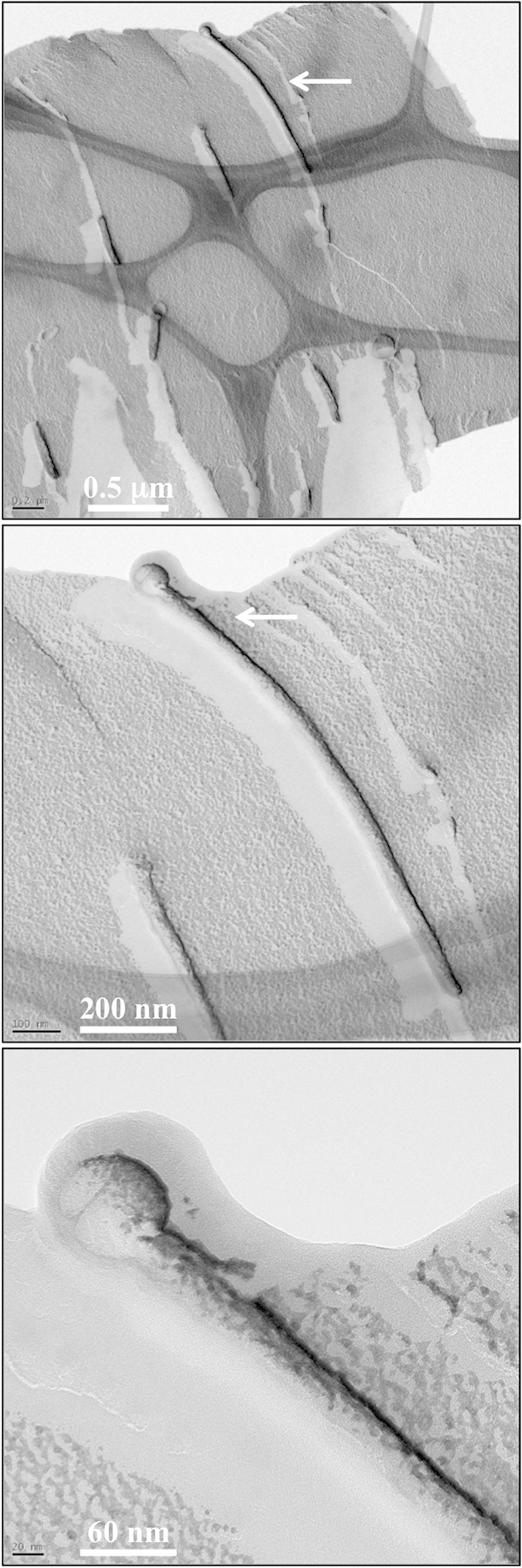
Examining renal epithelial cilia with HPF/FFTEM. Once a suitable structural configuration is selected (**top panel**), a higher magnification can be used for analysis (**middle panel**). To further understand the ultrastructure of the ciliary bulb, we could increase the magnification and image visibility (**lower panel**). Shown here is a typical ciliary bulb, which indicates that the bulb membrane is continuous from the cilia bilayer and that particle indentions are observed in the ciliary membrane and bulb. Arrow indicates a cilium to be analyzed

**Figure 4:**
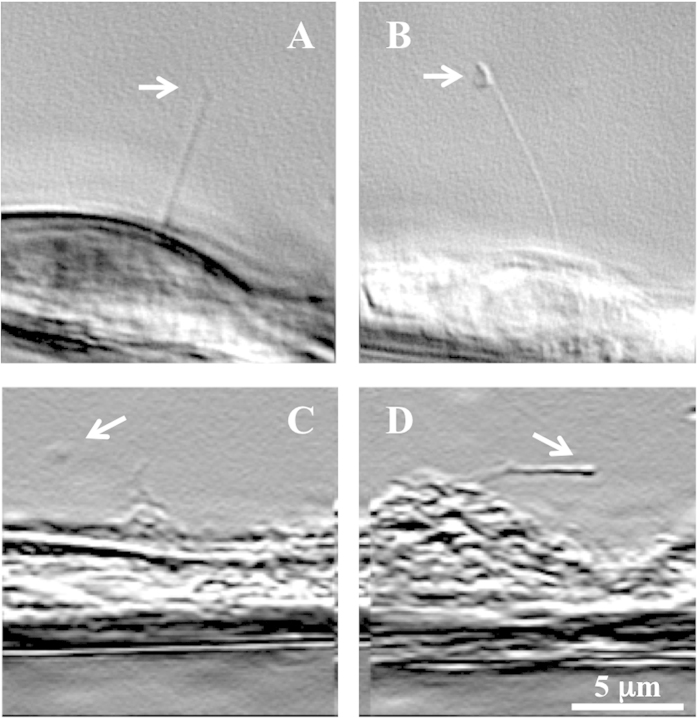
Effect of chemical fixation on ciliary structure. Shown here are live images of renal primary cilia in non-treated controls (**a,b**) and paraformaldehyde-treated cells (**c,d**). A nice, smooth elongated cilium was observed in the controls (without treatment). Treatment with 4% paraformaldehyde for 5 minutes grossly damages the structural integrity of the cilium. After treatment, the cilium seems to be weakened and is not projecting up straight. All images were taken from the side-view with high-resolution differential interference contrast (DIC) microscope (Nikon TiU). Cells were then grown on collagen-coated tungsten wire to allow cilia to orient outward for visualization, as previously described^18^.
