# Supplementary material for: Chemical-Free Technique to Study the Ultrastructure of Primary Cilium
Source: Sci Rep. 2015 Nov 2;5:15982. doi: 10.1038/srep15982 (PMC4629161; doi:10.1038/srep15982)

Chemical-Free Technique to Study the Ultrastructure of Primary Cilium

Ashraf M. Mohieldin, Wissam A. AbouAlaiwi, Min Gao, Surya M. Nauli

Department of Biomedical & Pharmaceutical Sciences, Chapman University School of Pharmacy, Irvine, CA 92618
Department of Urology, University of California at Irvine Medical Campus, Orange, CA 92868
Department of Medicinal & Biological Chemistry, University of Toledo School of Pharmacy, Toledo, OH 43614
Liquid Crystal Institute, Kent State University, Kent, OH 44242.

Corresponding:
Surya M. Nauli
Chapman University, School of Pharmacy
University of California at Irvine, Urology
9401 Jeronimo Road
Irvine, CA 92618-1908
Tel: 714-516-5485
Fax: 714-516-5481
Email: nauli@chapman.edu
Email: snauli@uci.edu

Supplemental Figure 1

The following 6 HPF/FFTEM micrographs show the presence of primary cilia. Cilia were pseudocolored in green.

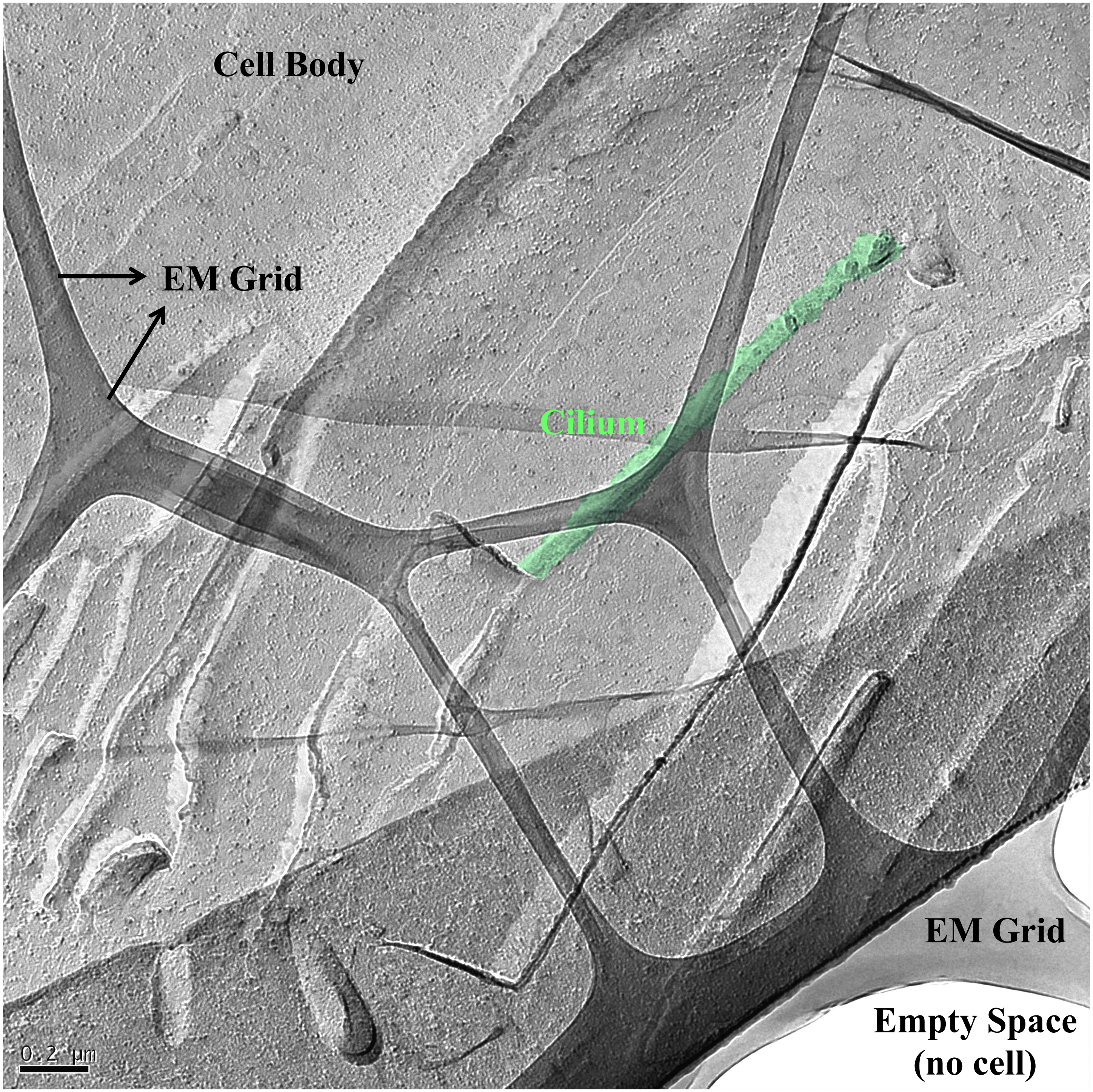

Cell Body
EM Grid
Cilium
EM Grid
Empty Space
(no cell)
0.2 µm

1.2

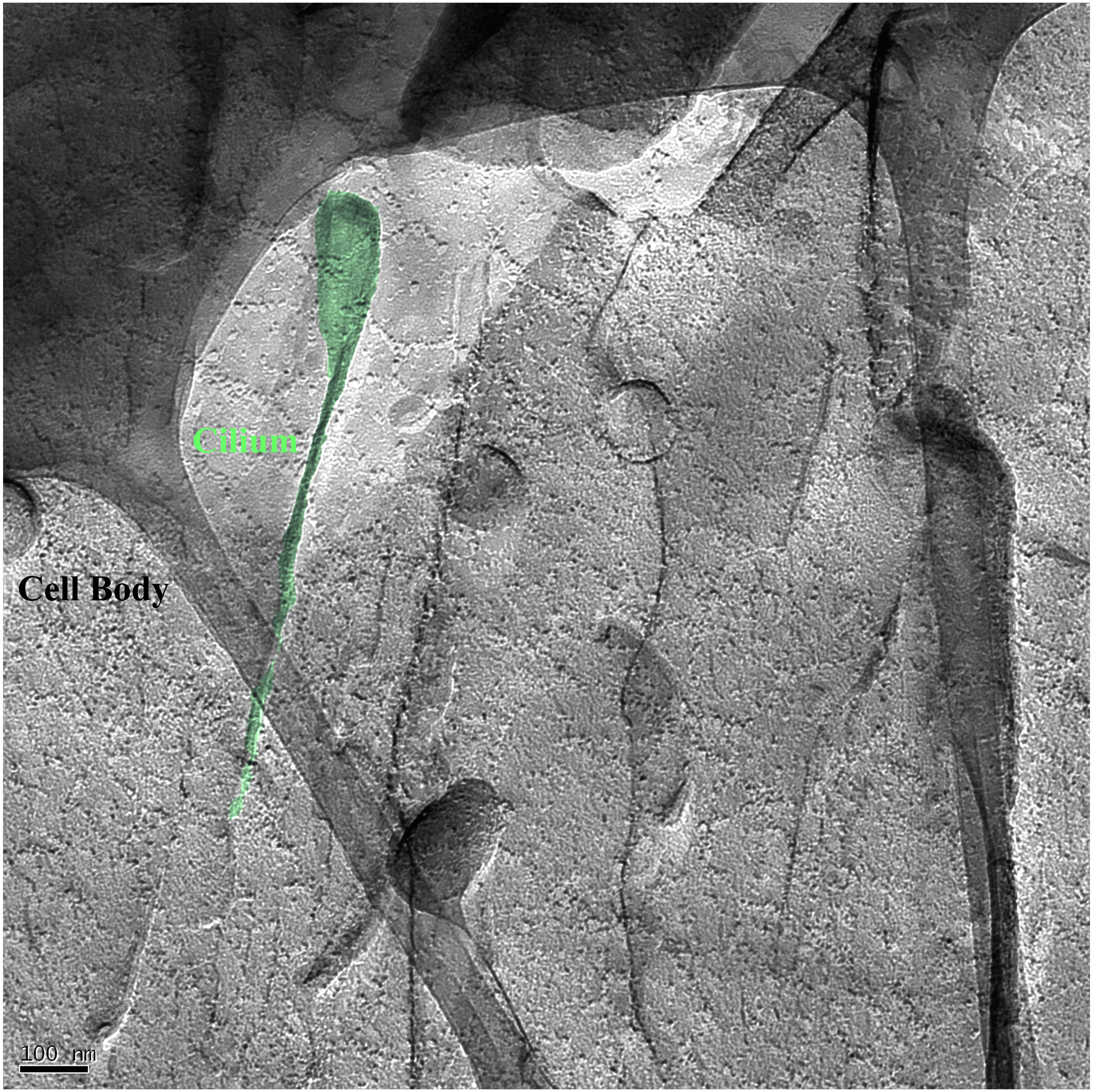

1.3

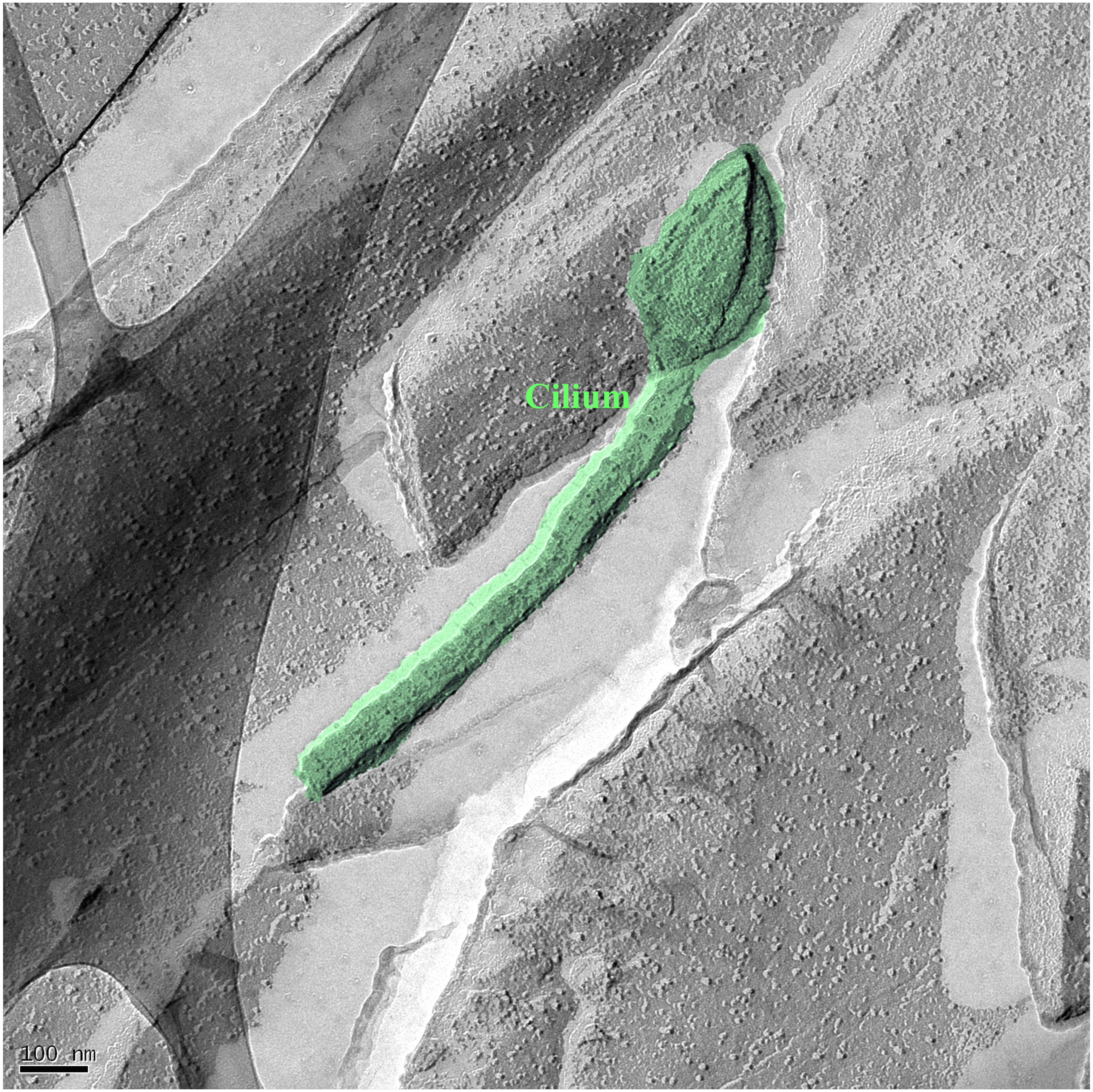

1.4

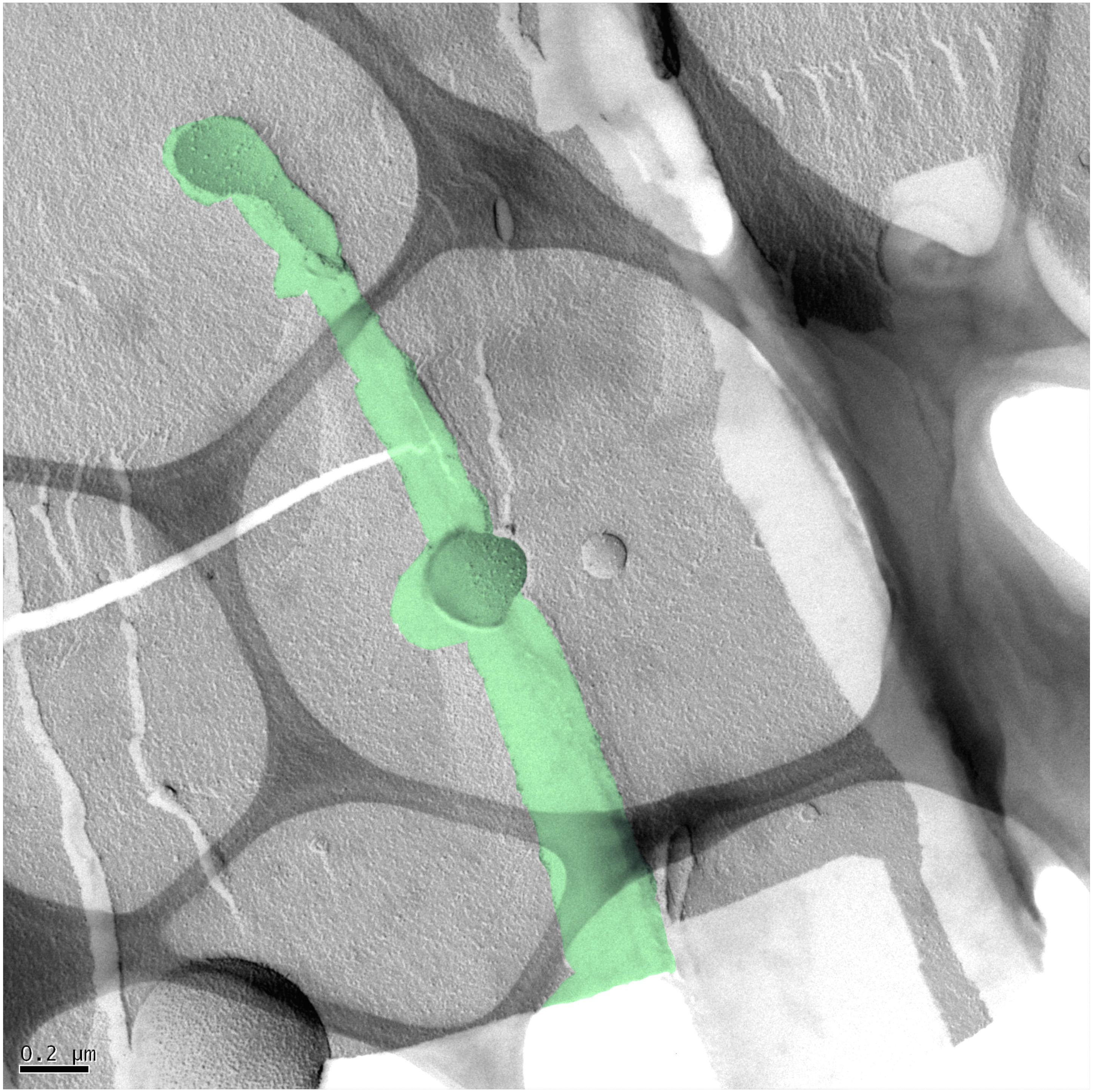

1.5

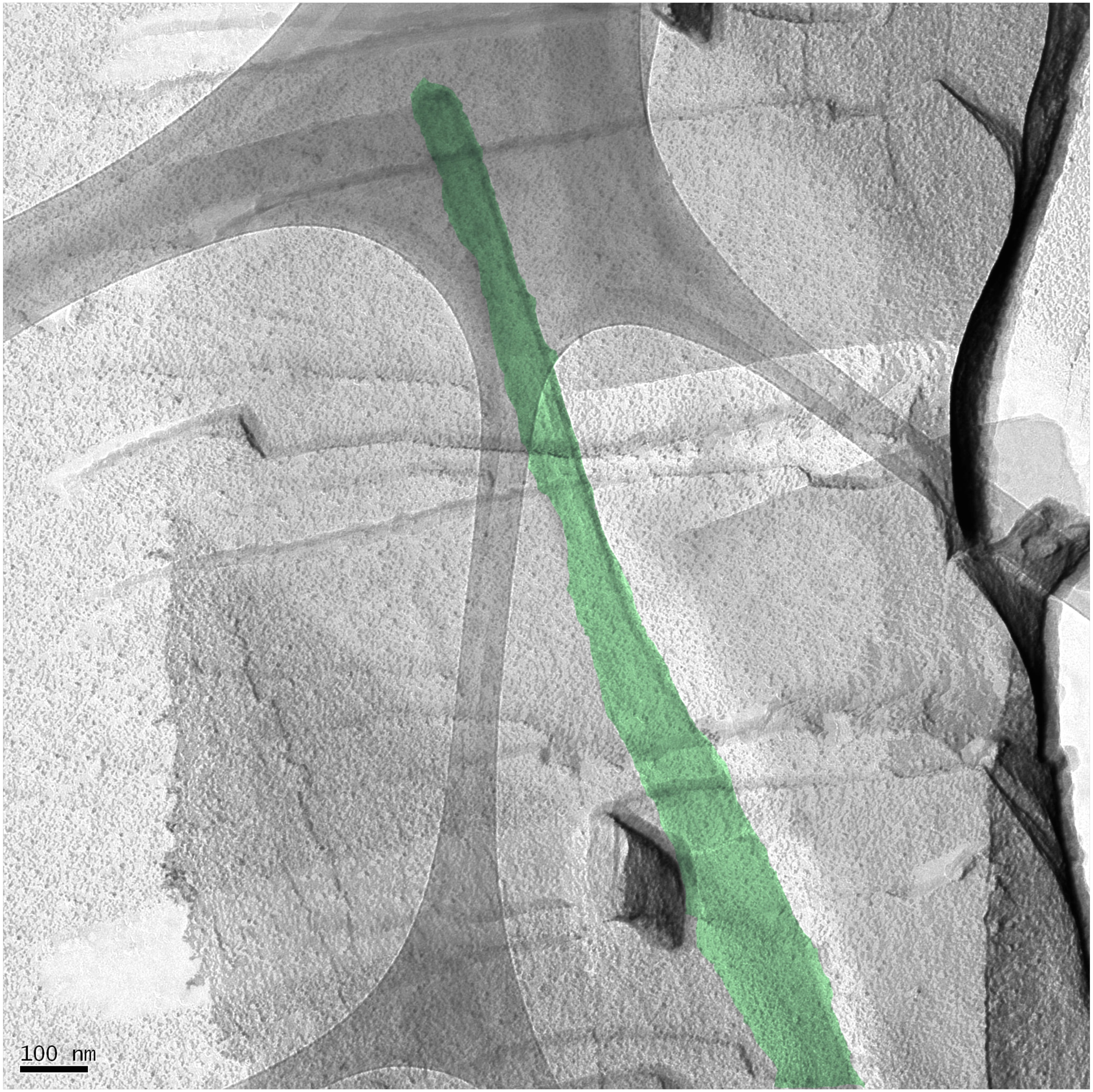

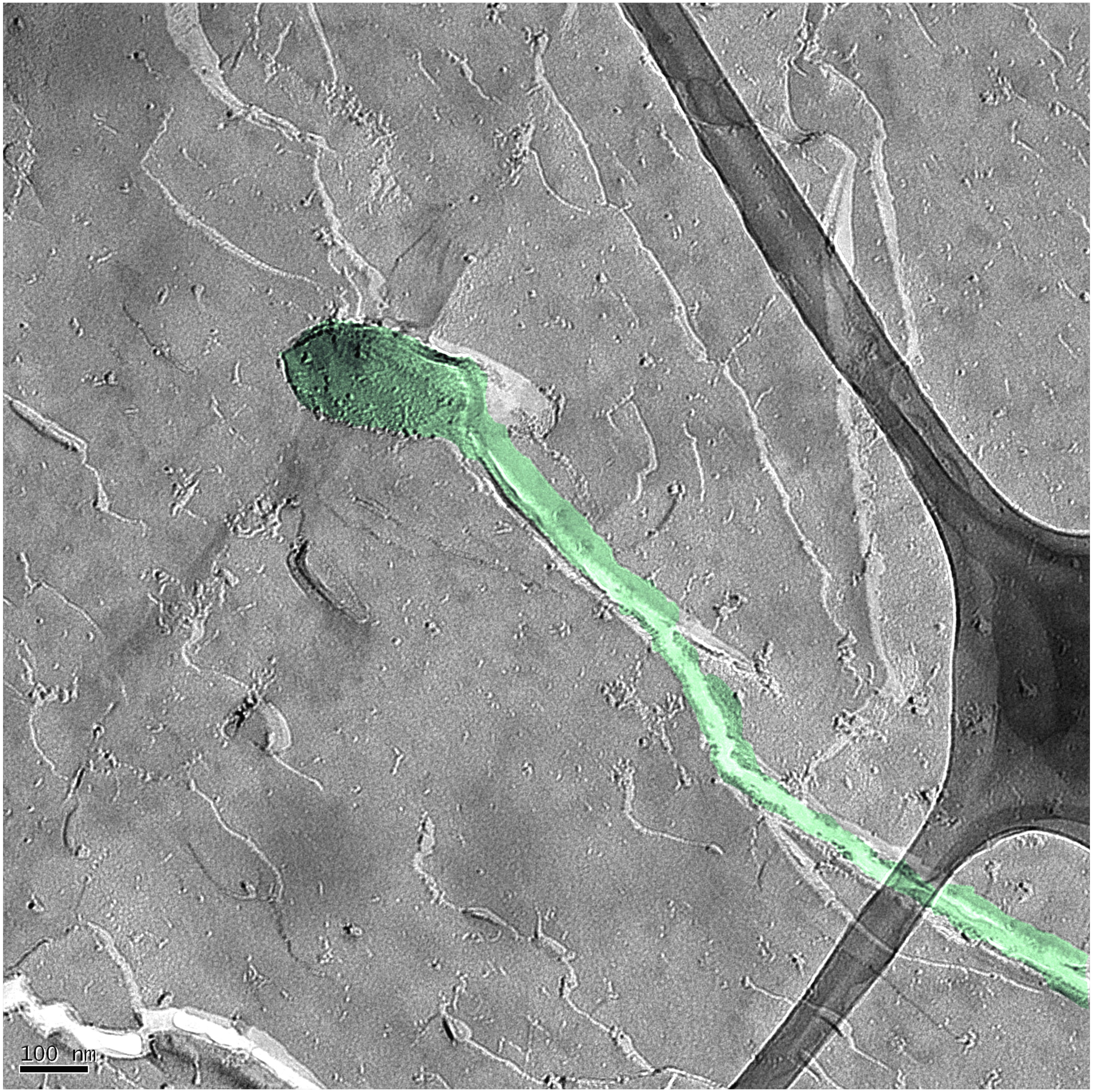

1.6

Supplemental Figure 2

The following 6 HPF/FFTEM micrographs show the representative images in which primary cilia are not observed.

2.1

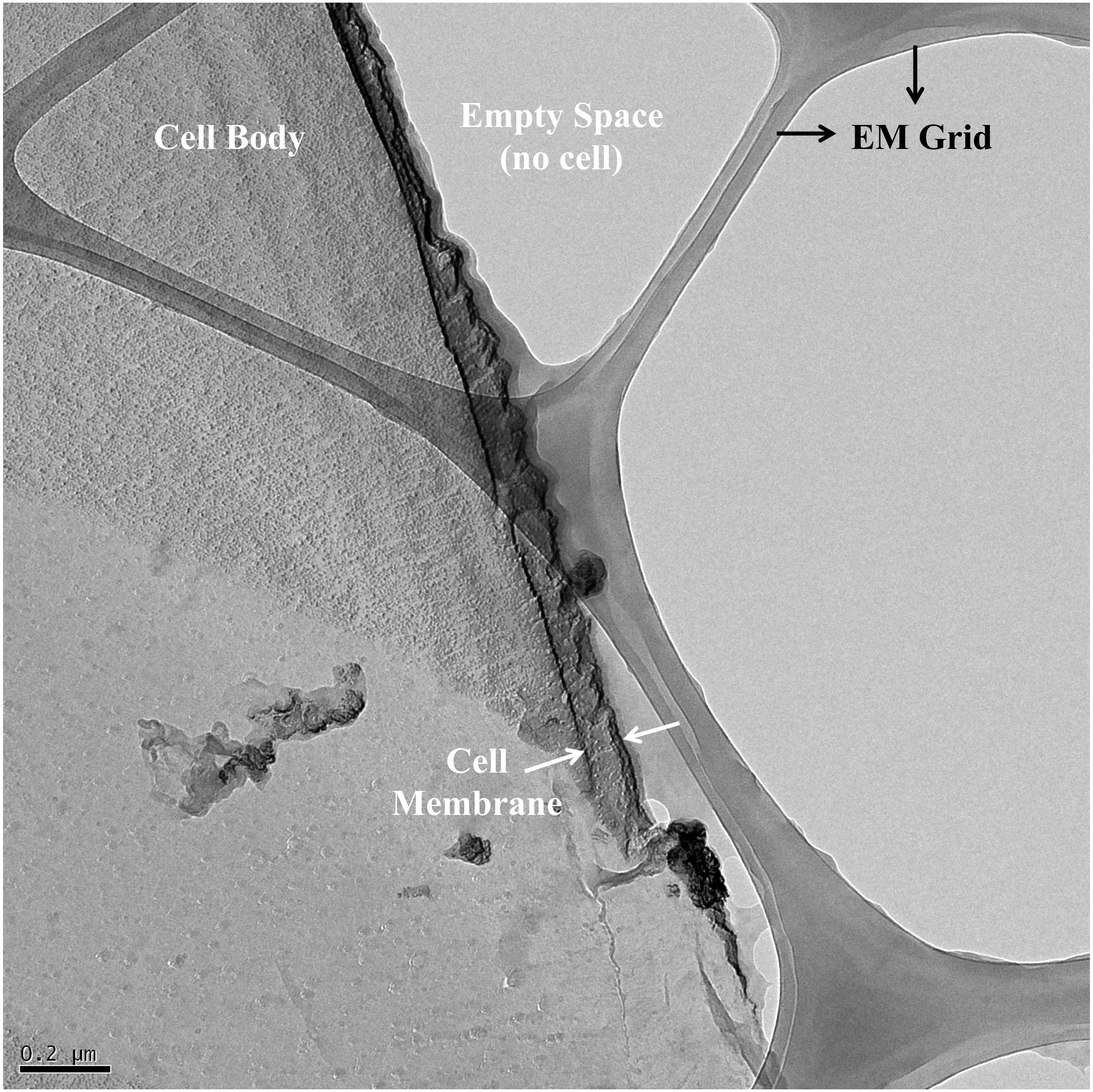

2.2

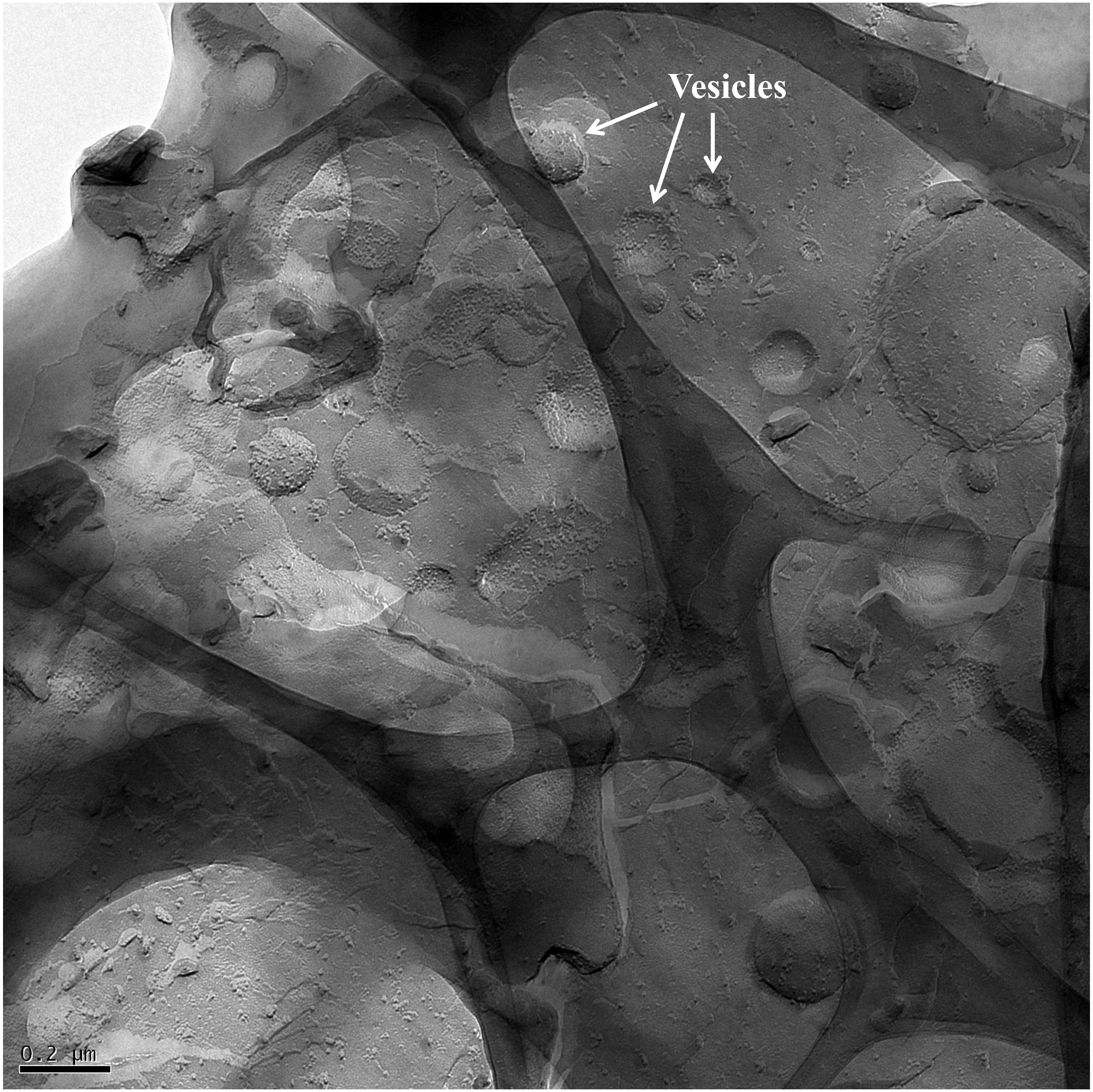

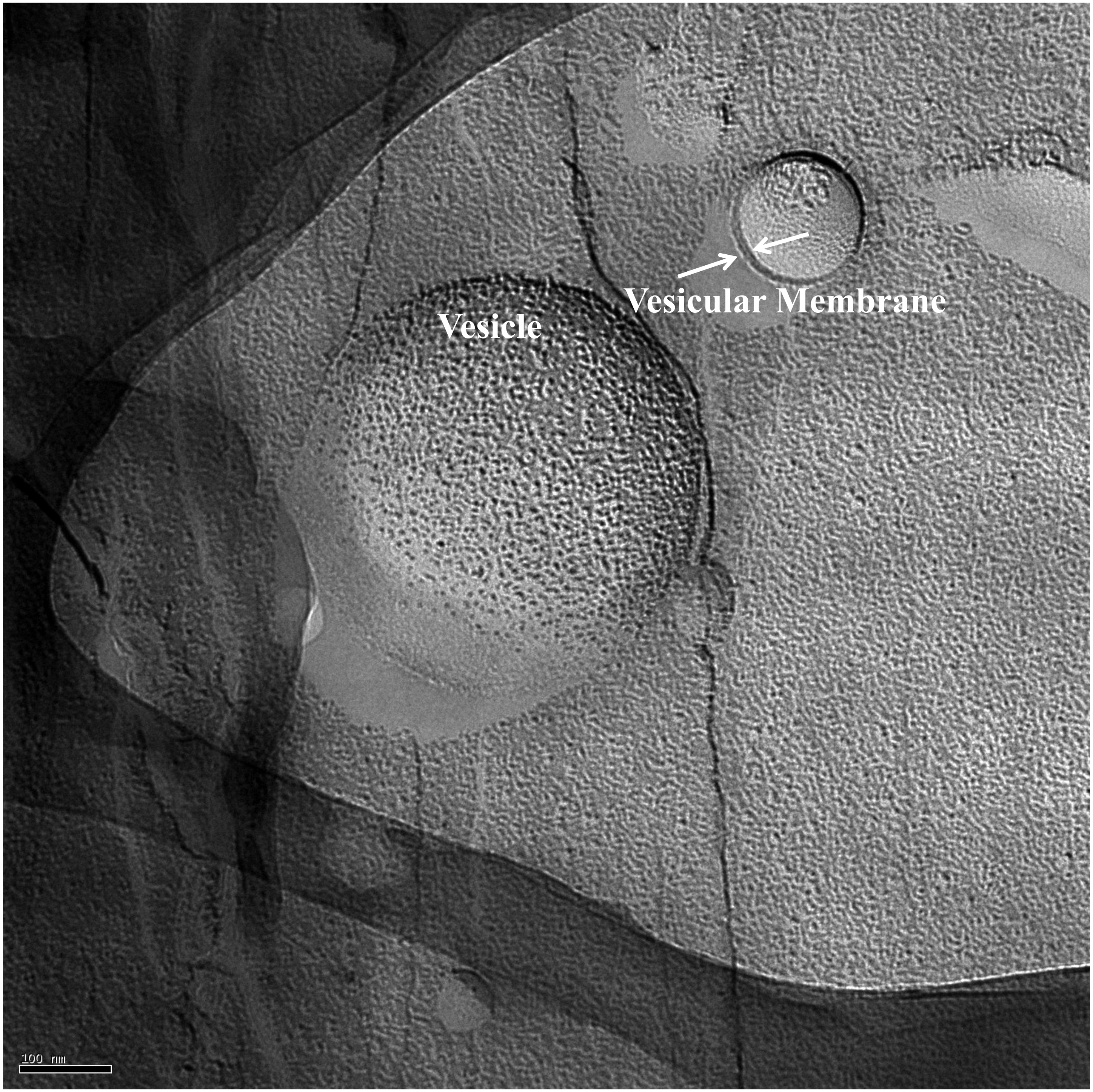

2.3

2.4

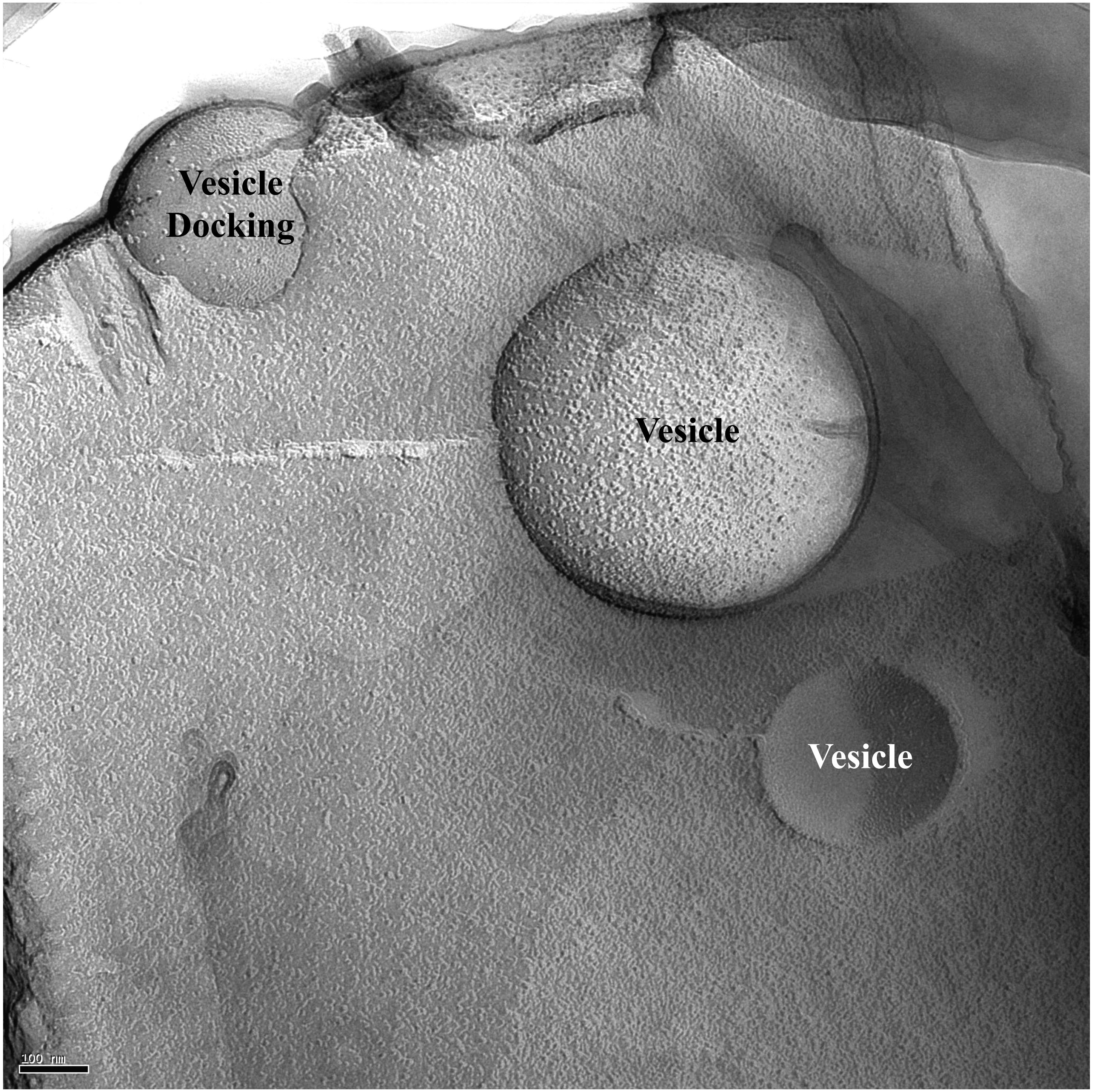

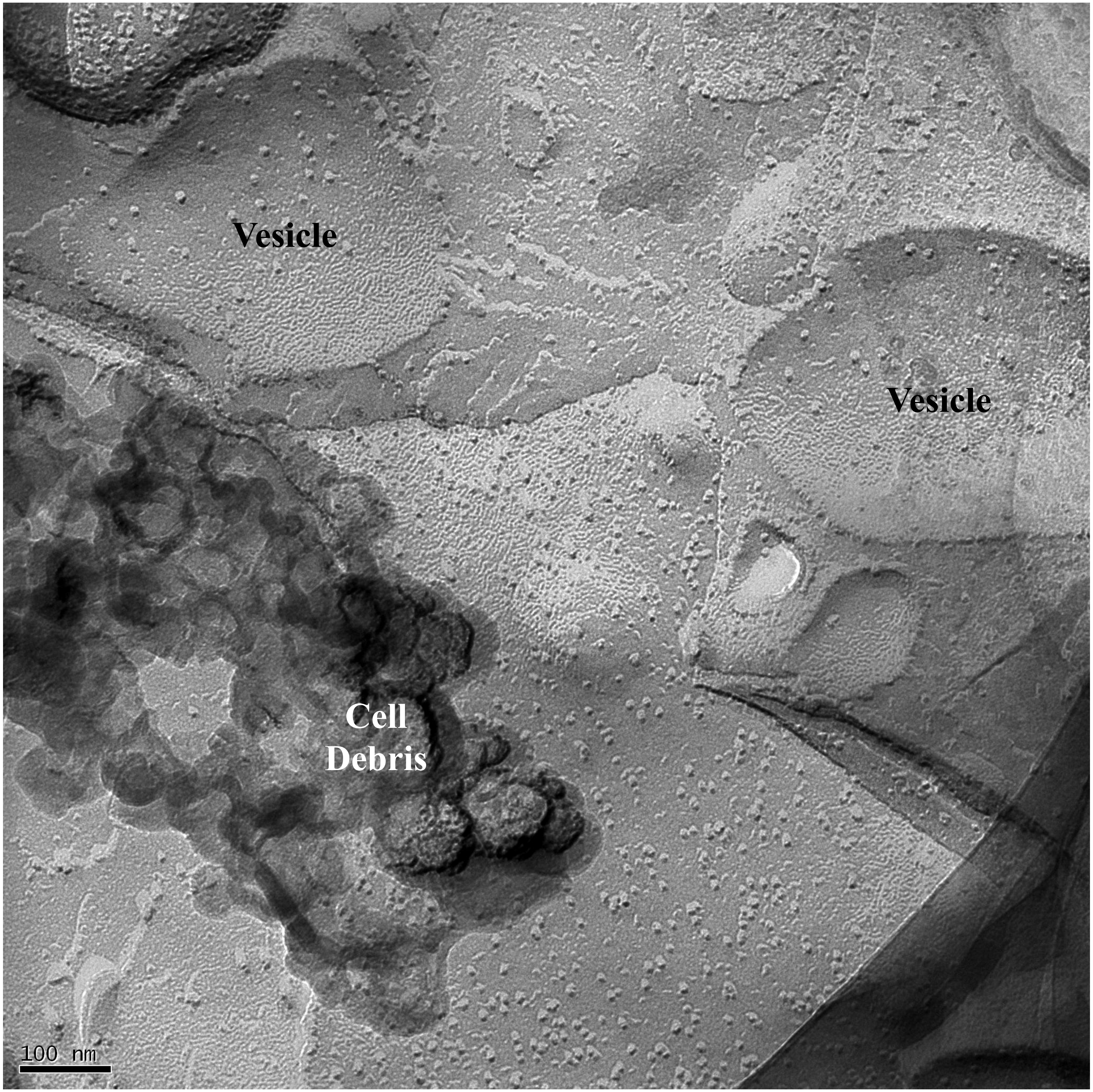
Vesicle
Vesicle
Cell
Debris
100 nm

2.6

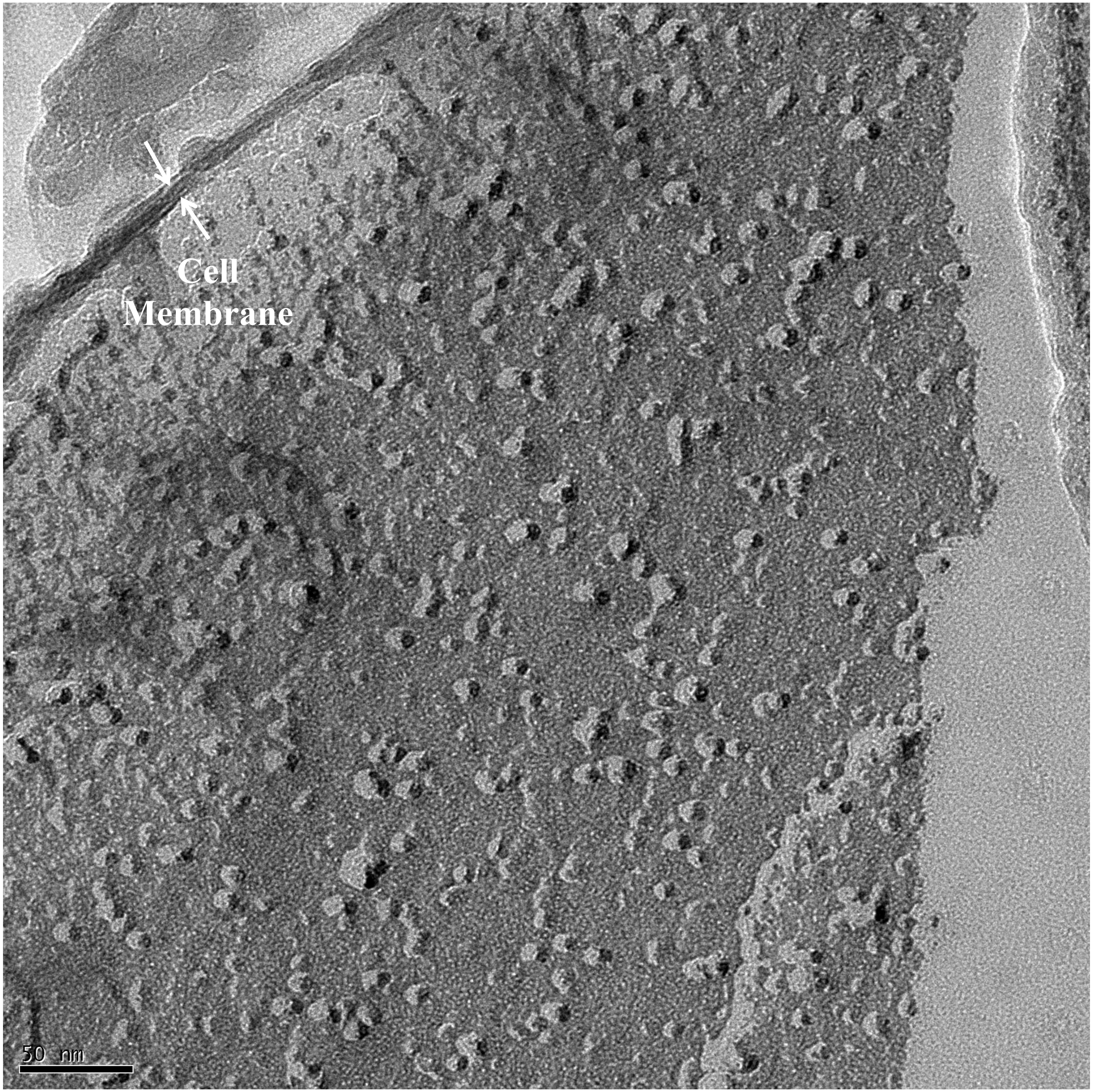

Supplement: Supplementary Data [file srep15982-s1.pdf]
